# Deep learning architectures for EEG-based classification of Dravet syndrome: A comparative study of pre-trained and non-pretrained hybrid CNN-LSTM models

**DOI:** 10.1371/journal.pone.0352991

**Published:** 2026-07-31

**Authors:** Sikandar Hussain, Soyiba Jawed, Ali Mir, Mona Ibrahim Ali, Raidah Al-Baradie, Norah Alaqili, Eman Mohammed Nassim Ali, Shahid Bashir

**Affiliations:** 1 Department of Computer and Software Engineering, College of Electrical and Mechanical Engineering, National University of Sciences and Technology(NUST), Islamabad, Pakistan; 2 Department of Pediatric Neurology, King Fahad Specialist Hospital, Dammam, Saudi Arabia; 3 Department of Adult Neurology, King Fahad Specialist Hospital, Dammam, Saudi Arabia; 4 Research Center, King Fahad Specialist Hospital Dammam, Dammam, Saudi Arabia; Georgia State University, UNITED STATES OF AMERICA

## Abstract

**Objective:**

This study explores the potential of artificial intelligence (AI) using a hybrid deep learning Convolutional Neural Network–Long Short-Term Memory (CNN-LSTM) framework, for EEG-based classification and analysis of Dravet Syndrome (DS).

**Method:**

The study cohort comprised nine pediatric patients with DS, confirmed through either a heterozygous pathogenic mutation in the SCN1A gene or a clinical diagnosis consistent with established diagnostic criteria. In addition, EEG recordings from age-matched healthy control subjects and pediatric patients with non-Dravet epilepsy (“abnormal” EEG) were included. Data on demographic information, seizure characteristics, developmental skills, cognitive functions, and genetic results were gathered from patient records. EEG recordings were analyzed using a subject-independent leave-one-subject-out validation strategy, spatial and temporal features by employing this model on preprocessed EEG data, effectively differentiating DS patients from Abnormal cases and healthy controls.

**Result:**

Among the evaluated CNN-LSTM models, the pre-trained architecture achieved superior performance with improved stability across most subjects, with an overall accuracy of 85%, balanced accuracy of 85%, a macro-averaged F1-score of 0.85, and a macro-averaged ROC–AUC of 0.87, demonstrating stable performance for multi-class EEG classification of DS, Abnormal, and control subjects. The non-pretrained model showed reduced sensitivity and increased inter-class confusion, particularly for DS and Abnormal classes.

**Conclusion:**

This study demonstrates that a pre-trained CNN-LSTM framework can support automated EEG-based classification of DS-related patterns as a proof-of-concept methodological approach, even in the context of limited subject availability. EEG-specific pretraining improves classification consistency and feature separability compared with training from scratch, highlighting the value of representation learning for rare epilepsy syndromes. Larger multi-center datasets and prospective validation will be required to assess robustness, generalizability, and clinical utility.

## 1. Introduction

Dravet Syndrome (DS) originally termed Severe Myoclonic Epilepsy of Infancy (SMEI), was initially identified in France by pediatric epileptologist Charlotte Dravet in 1978 and later renamed in her honor in 1989 [1]. It is a rare, early-onset epileptic encephalopathy marked by severe seizures and developmental delays. Typically, DS presents within the first year of life, with seizures frequently triggered by fever, illness, or temperature fluctuations, resulting in cognitive and motor challenges [2,3]. Diagnosis is primarily clinical, based on the onset of characteristic seizures typically appearing between 2 and 15 months of age [4], often requiring differentiation from other epilepsy syndromes [5].

DS is most frequently associated with pathogenic variants in the SCN1A gene, which encodes the alpha subunit of the voltage-gated sodium channel Nav1.1. SCN1A mutations are identified in approximately 80–85% of individuals with DS, while a minority of cases are diagnosed clinically despite negative genetic findings [2,6,7]. Genetic confirmation, when available, supports diagnostic accuracy and facilitates differentiation from other epilepsy syndromes with overlapping clinical and EEG features [8].

Despite significant advancements in genetic diagnostics, there remains a lack of accurate, scalable, and non-invasive tools for early functional assessment and longitudinal monitoring of DS, particularly in resource-limited or acute-care settings. In this context, EEG-based deep learning methodology has emerged as a promising approach demonstrating potential for real-time analysis directly from EEG signals with minimal processing [9]. Unlike traditional approaches that require extensive feature engineering, this strategy relies on minimally conditioned EEG data, applying only essential scaling to preserve the physiological characteristics of the raw signals prior to model input [10]. However, EEG patterns in DS can overlap with other epilepsy syndromes, making automated and objective classification particularly challenging.

Here, a hybrid Convolutional Neural Network–Long Short-Term Memory (CNN-LSTM) network is proposed for EEG-based signal analysis. The CNN is responsible for extracting spatial patterns from EEG signals [9], while the LSTM captures temporal patterns from EEG signal sequences [11].

The proposed framework is evaluated under an offline analysis setting. Future extensions may explore adaptation of this approach toward near-real-time EEG monitoring and longitudinal pattern analysis, particularly for individualized disease tracking and decision support [12,13].

## 2. Methodology

### 2.1. Study design and participants

A retrospective analysis of medical records was conducted to examine individuals diagnosed with DS. The study cohort consisted of nine pediatric patients with genetically confirmed DS who received care at King Fahad Specialist Hospital – Dammam (KFSHD) in Saudi Arabia, between 2009 and 2023. This retrospective case series focused on clinical, electrophysiological, and genetic features of DS followed at the center.

### 2.2. Ethical considerations

This study strictly adheres to ethical guidelines and received approval from the Institutional Review Board at King Fahad Specialist Hospital – Dammam.

### 2.3. Subjects

In this cohort, nine patients with DS were identified. Among them, eight patients (85.7%) carried a heterozygous pathogenic mutation in the SCN1A gene, while one patient (14.3%) was clinically diagnosed despite negative genetic testing. All patients exhibited epilepsy and developmental delay, with seizures as the presenting symptom in each case. The age of seizure onset ranged from 2.5 to 30 months, with a mean onset age of 7.04 months. Most of the patients (55.5%, n = 5) presented within 4–6 months of age, while one patient (11.1%) exhibited seizure onset at 2.5 months. Additionally, two patients (22.2%) experienced seizure onset between 7–9 months, and one patient (11.1%) had a delayed onset at 30 months. The abnormal epilepsy group included five patients (n = 5) with heterogeneous epilepsy phenotypes not meeting criteria for DS. This group comprised four females and one male, with ages ranging from 2.8 to 7 years.

The control group consisted of ten healthy individuals (n = 10) without a history of neurological or psychiatric disorders. The mean age of controls was approximately 10.1 years (range: 6.5–13.8 years), including five males and five females.

### 2.4. Data collection

Clinical records were systematically reviewed, including demographic data, seizure characteristics, developmental milestones, and treatment histories. All patients underwent EEG, interpreted by pediatric epileptologists (AM, RA). Genetic testing was performed to confirm the diagnosis of Dravet syndrome and to support accurate cohort classification. Molecular analysis was conducted using next-generation sequencing–based epilepsy gene panels or whole exome sequencing, with pathogenic and likely pathogenic variants classified according to the American College of Medical Genetics and Genomics (ACMG) guidelines [14]. Where indicated, segregation analysis was performed in available family members.

### 2.5. Study objectives

**Fig 1:**
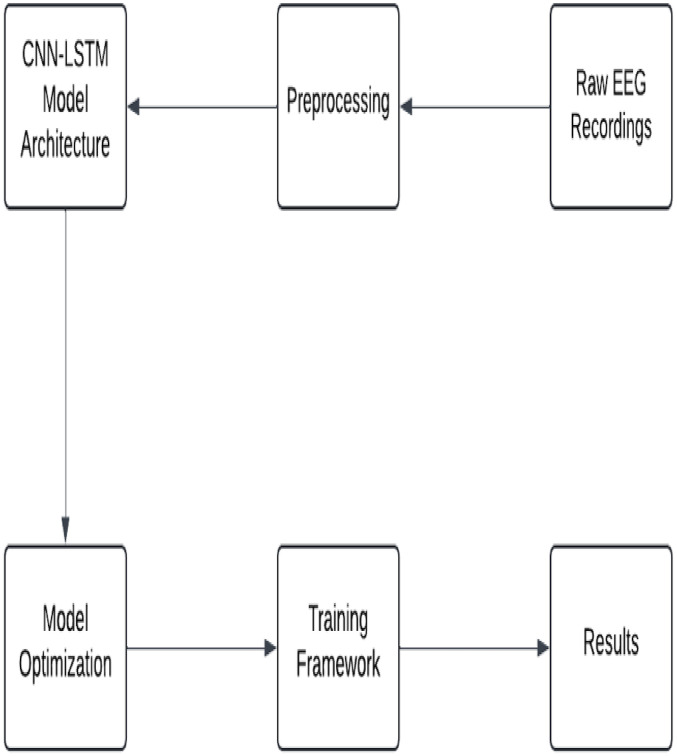
The pipeline delineates a comprehensive approach to efficiently classify and analyze EEG signals. The process begins with the acquisition of raw EEG recordings, which are then subjected to a series of preprocessing steps. The resulting processed data is subsequently inputted into a convolutional neural network and long short-term memory (CNN-LSTM) model architecture. The CNN is employed to extract spatial features, while the LSTM component focuses on capturing temporal dependencies within the data. Following the architecture setup, the model is then optimized and trained, leading to results that substantiate the model’s efficacy in classifying EEG signals accurately.

The aim of this study was to apply deep learning on a minimally preprocessed EEG dataset to differentiate between age-matched control pediatric participants (N = 10), DS patients (N = 9) and abnormal (N = 5). From an initial pool of 20 control subjects, a subset of 10 controls was selected at the subject level to mitigate class imbalance relative to the patient groups. The raw EEG data underwent a structured conditioning pipeline that included Cz-referenced montage using Cz as a common reference electrode, segmentation into fixed-length segments, removal of duplicate entries, and channel-wise normalization to eliminate amplitude variability while preserving the temporal and physiological characteristics of the signal. The dataset was meticulously curated to ensure comparability between the three groups, with cohorts matched by age to eliminate potential confounding variables. The overall workflow of the EEG classification pipeline is illustrated in Fig 1.

### 2.6. Preprocessing

All preprocessing was conducted using a Kaggle notebook. This study utilizes EEG recordings (EDF files) from three subject groups: DS, healthy controls, and abnormal. Rather than using a common-reference montage, a Cz-referenced electrode configuration (F3–Cz, Fz–Cz, F4–Cz, C3–Cz, C4–Cz, P3–Cz, Pz–Cz, P4–Cz), was applied to enhance signal clarity and minimize artifacts [14]. Further, to reduce the influence of muscle artifacts, specifically the contribution from the temporal muscle artifacts in the gamma frequency band, eight electrode locations were selected for further analysis. This electrode selection ensured adequate spatial coverage of the brain regions. To increase the effective number of training samples and support stable model training, the dataset was segmented into 50% overlapping windows, containing 5000 samples each and removing duplicate entries. Since this study focuses on multi-class classification (of DS vs. healthy controls vs abnormal), each segment was labeled as ‘1’ for DS samples, ‘0’ for the control group and 2 for abnormal.

To prevent data leakage across EEG segments from the same subject, model evaluation was performed using a Leave-One-Subject-Out (LOSO) cross-validation strategy. In each iteration, all EEG segments from one subject were held out as the test set, while EEG segments from the remaining subjects were used for model training and validation. This procedure ensured that no subject contributed data to more than one split and that performance metrics reflect subject-level generalization. For each LOSO fold, predictions were generated for all EEG segments belonging to the held-out subject. To compute overall performance metrics, including the confusion matrix, segment-level predictions from all test folds were aggregated (pooled) across subjects. Although pooled predictions were used for reporting, the evaluation remained subject-independent, as all segments from each subject were evaluated exclusively within their respective LOSO fold.

A total of nine EDF files were analyzed for the Abnormal Epilepsy group. These recordings correspond to multiple EEG sessions acquired at different time points from five patients. To prevent pseudo-replication, recordings from each patient were grouped together and treated as a single subject during LOSO validation. This approach ensured that all recordings from the same patient were held out together in each test fold, preventing data leakage while enabling methodological validation of the framework. Accordingly, the results should be interpreted as proof-of-concept validation of the proposed ML/AI framework rather than as population-level generalization.

**Fig 2:**
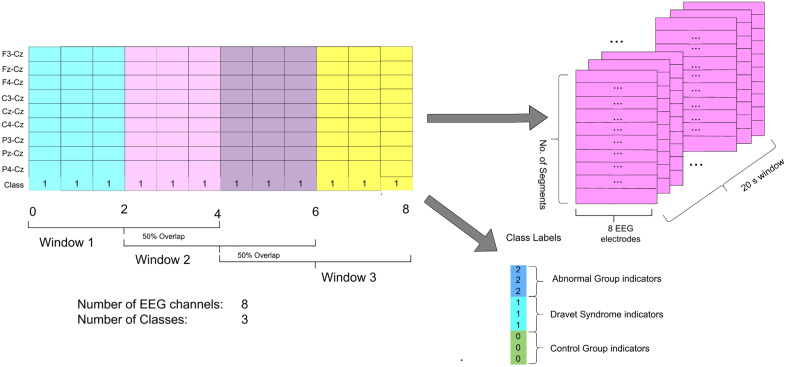
EEG signals were recorded at a sampling frequency of 250 Hz. For analysis, the continuous recordings were divided into 50% overlapping segments of 5000 data points, which correspond to approximately 20 seconds of EEG activity per segment (5000 ÷ 250 = 20 s). Data from eight electrodes (F3-Cz, Fz-Cz, F4-Cz, C3-Cz, C4-Cz, P3-Cz, Pz-Cz, and P4-Cz) were included. In total, 9,897 segments were obtained, each containing signals from the eight channels over 5000 time points making a three-dimensional matrix of size (9,897, 8, 5000), that serves as input for deep learning model. A corresponding label was assigned to each segment to indicate whether it originated from a DS or Abnormal patient or a control subject.

For the model, the input shape of each EEG segment was (5000, 8, 1), where 5000 represents the number of sample points per segment, 8 corresponds to the actual number of electrodes and 1 represents a single input shown in Fig 2. The output labels were represented as integer class indices corresponding to Control (0), DS (1), and abnormal (2) consistent with the multi-class classification setup, and optimized using a cross-entropy loss function with integer class labels [10,15]. Normalization was applied on the data by using a StandardScaler technique of the scikit-learn library to eliminate amplitude variability across channels. Each channel was scaled to have a mean of 0 and a standard deviation of 1 by this technique.

### 2.7. Deep learning-based classification model

Following normalization, the processed data was utilized to develop deep learning-based framework for behavioral modeling. All the implementation was done in Python, utilizing PyTorch deep learning framework [16,17]. Given the substantial computational requirements, all the implementation was executed in Kaggle Notebooks, which provides access to powerful GPU resources, specifically the NVIDIA P100 GPU, to accelerate model training.

Two CNN-LSTM architectures were evaluated in this study. The first model employed a custom CNN trained from scratch and combined with an LSTM layer for temporal modeling. The second model used a pre-trained ShallowFBCSPNet as the CNN feature extractor, followed by an LSTM layer for sequence modeling. This design enabled a direct comparison between non-pretrained and pre-trained CNN-LSTM architectures under identical training and evaluation conditions.

**Fig 3:**
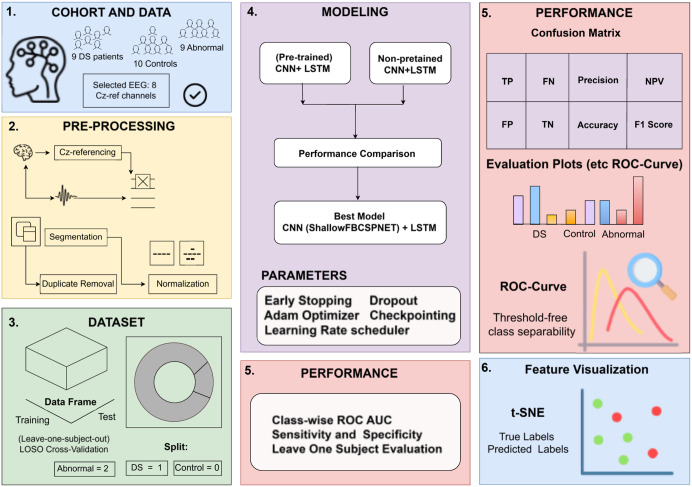
Schematic representation of the EEG processing and classification workflow used in this study. EEG channels are first selected and normalized to reduce amplitude variability across recordings. The continuous EEG data are then divided into overlapping segments, which are provided as input to a CNN-LSTM model. The convolutional layers learn spatial patterns from the EEG channels, while the LSTM layers capture temporal relationships within each segment. Model training includes checkpointing, learning rate adjustment, and early stopping to improve training stability. Model performance is evaluated using subject-independent validation and ROC-based metrics, while t-SNE is employed for feature representation visualization. The figure summarizes the key stages of EEG segmentation, model training, performance evaluation, and feature visualization.

Both architectures integrate convolutional neural networks for spatial feature extraction from multichannel EEG signals with LSTM layers for modeling temporal dependencies across time. The first architecture uses a custom CNN trained from scratch as shown in Table 1, while the second employs a pre-trained ShallowFBCSPNet as the CNN feature extractor as shown in Table 2. This design enables a focused comparison between pre-trained and non-pretrained CNN backbones under identical training and evaluation conditions. CNN-LSTM architectures have been previously reported to effectively capture joint spatial–temporal patterns in EEG signals [18], and their applicability to neurophysiological signal analysis has been demonstrated in real-time EEG-based cognitive state classification tasks [9]. The overall architecture used in this work is illustrated in Fig 3.

**Table 1:** Architecture of the non-pretrained CNN–LSTM model.

Block	Layer (Type)	Output Shape	Param #
Input	EEG Input	(Batch, 8, 5000)	0
CNN-Block 1	Conv1D (64, k = 3)	(Batch, 64, 5000)	1,792
	BatchNorm + LeakyReLU	(Batch, 64, 5000)	128
	MaxPool1D (2)	(Batch, 64, 2500)	0
	Dropout (0.3)	(Batch, 64, 2500)	0
CNN-Block 2	Conv1D (128, k = 3)	(Batch, 128, 2500)	24,704
	BatchNorm + LeakyReLU	(Batch, 128, 2500)	256
	MaxPool1D (2)	(Batch, 128, 1250)	0
	Dropout (0.3)	(Batch, 128, 1250)	0
CNN-Block 3	Conv1D (256, k = 3)	(Batch, 256, 1250)	98,560
	BatchNorm + LeakyReLU	(Batch, 256, 1250)	512
	MaxPool1D (2)	(Batch, 256, 625)	0
	Dropout (0.3)	(Batch, 256, 625)	0
Reshape	Permute	(Batch, 625, 256)	0
Temporal	LSTM (2 layers, h = 64)	(Batch, 625, 64)	164,864
	Last Time Step	(Batch, 64)	0
FC-Block	Dense (64 → 256)	(Batch, 256)	16,640
	ReLU + Dropout (0.5)	(Batch, 256)	0
	Dense (256 → 128)	(Batch, 128)	32,896
	ReLU + Dropout (0.4)	(Batch, 128)	0
Output	Dense (128 → 3)	(Batch, 3)	387

Table above lists the layers of the CNN-LSTM model used for EEG classification and their output dimensions and parameter count.

**Table 2:** Architecture of the pretrained CNN–LSTM model.

Block	Layer (Type)	Output Shape	Param #
Input	EEG Input	(Batch, 8, 5000)	0
CNN Backbone (Pretrained)	Temporal Conv (ShallowFBCSPNet)	(Batch, 40, 5000)	Pretrained
	Spatial Conv (Depthwise)	(Batch, 40, 5000)	Pretrained
	BatchNorm	(Batch, 40, 5000)	Pretrained
	Square Nonlinearity	(Batch, 40, 5000)	0
	Avg Pooling	(Batch, 40, ~ 61)	0
	Log Activation	(Batch, 40, ~ 61)	0
	Dropout	(Batch, 40, ~ 61)	0
Reshape	Squeeze + Permute	(Batch, 61, 40)	0
Temporal Modeling	LSTM (1 layer, h = 64)	(Batch, 61, 64)	27,904
	Last Time Step	(Batch, 64)	0
FC-Block	Dense (64 → 128)	(Batch, 128)	8,320
	ReLU + Dropout (0.5)	(Batch, 128)	0
Output	Dense (128 → 3)	(Batch, 3)	387

Architecture of the pre-trained CNN-LSTM model using ShallowFBCSPNet as the CNN backbone. The table details the layer-wise configuration, output dimensions, and parameter counts for the pre-trained CNN-LSTM architecture. Pre-trained layers are indicated accordingly.

#### 2.7.1. Input data for CNN layers.

**Fig 4:**
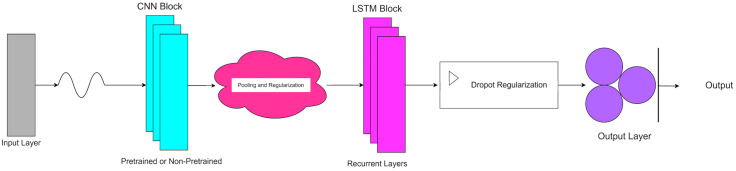
The figure shows architecture of the hybrid CNN-LSTM model used for EEG signal classification. The input EEG data was provided as a three-dimensional tensor. The CNN block (pre-trained and non-pretrained) uses convolutional layers to extract spatial features which are processed through LeakyReLU activation and batch normalization and max pooling. The extracted spatial features were then passed to a long short-term memory (LSTM) block to model temporal dependencies in the EEG sequences. The LSTM block consists of stacked recurrent layers with dropout regularization to reduce overfitting. The system uses a fully connected output layer which employs softmax activation to generate class probabilities for DS, control and Abnormal groups.

The EEG data are represented as a three-dimensional matrix, as illustrated in Fig 4. Before being fed into the CNN, this matrix is treated as a tensor, which allows it to be processed by convolutional layers. Following the input stage, a 1D convolutional layer with 256 filters and a kernel size of 3 is used to extract spatial features from the EEG signals. A LeakyReLU activation function with a negative slope of 0.1 is then applied, and batch normalization is used to stabilize the learning process.

#### 2.7.2. CNN layers.

Each CNN-LSTM architecture begins with a 1D convolutional layer with 256 filters and a kernel size of 3 to extract spatial features from the EEG signals. A leakyReLU activation function was applied with a negative slope of 0.1, followed by batch normalization to stabilize learning. To reduce feature dimensionality and computational load, MaxPooling1D layer with a pool size of 2 was used. This was followed by a dropout layer to prevent overfitting.


$$\begin{array}{c} {\text{y}}^{\left(\text{l}\right)}[\text{t}]=(\text{x}*\text{w})(\text{t})+\text{b}=\sum_{\text{a}=-\infty }^{\infty }\text{x}(\text{a})\text{w}(\text{t}-\text{a})+\text{b} \end{array}$$ (1)

${\text{y}}^{\left(\text{l}\right)}[\text{t}]$ is the output feature map at time t, x is the input tensor, w is the weight kernel filter, b is the bias, and * is the convolution operator.


$$\begin{array}{c} \text{LeakyReLU}(\text{z})=\;\left\{ \begin{array}{c} \text{z},\text{ if z }\geq \text{0}\\ \text{az},\text{ if z}<\text{0} \end{array}\right. \end{array}$$ (2)

Where z is the input to the activation function and α is the negative slope coefficient (typically 0.01 or 0.1).


$$\begin{array}{c} {\text{p}}_{\text{i}}=\underset{\text{j}\in \text{window}}{\text{max}}\left({\text{x}}_{\text{j}}\right) \end{array}$$ (3)

${\text{p}}_{\text{i}}$ is the output of the pooling window, and ${\text{x}}_{\text{j}}$ is the input values for pooling window.


$$\begin{array}{c} \text{y}=\text{r}⊙\text{ h} \end{array}$$ (4)

In Equation 5, r is the binary mask sampled from a Bernoulli distribution r ∼ Bernoulli(p), h is the input and ⊙ is the element wise multiplication.

In the pre-trained configuration, the convolutional layers were initialized using ShallowFBCSPNet weights, while in the non-pretrained configuration, all CNN layers were trained from scratch.

#### 2.7.3. LSTM layers.

After the CNN layer, LSTM layers are incorporated to capture the model’s temporal dependencies in the EEG signals. The first LSTM layer consists of 256 units with a tanh activation function to capture long-term dependencies in the data. This was followed by a LeakyReLU activation function and a 30% dropout. The second LSTM layer also contains 256 units and follows the same activation and dropout strategy. Finally, a third LSTM layer with 128 units consolidates the temporal features, followed by LeakyReLU activation and a 40% dropout layer for robust feature extraction. To better understand the role of these layers, it is important to note that an LSTM has three gates, first one is the Forget gate, which determines which information from the previous cell state should be retained or discarded.


$$\begin{array}{c} {\text{f}}_{\text{t}}=\;\sigma ({\text{W}}_{\text{f}}\;.\;\left[{\text{h}}_{\text{t}-\text{1}},\;{\text{ x}}_{\text{t}}\right]+\;{\text{b}}_{\text{f}} \end{array}$$ (5)

Where σ is the activation function, ${\text{W}}_{\text{f}}$ is the weight matrix, ${\text{b}}_{\text{f}}$ is the bias, ${\text{h}}_{\text{t}-\text{1}}$ is the previous hidden state, and${\text{ x}}_{\text{t}}$ is the current input of the LSTM.

Second gate is the Input gate, which determines which information from the current input ${\text{ x}}_{\text{t}}$ should be added to the cell state.


$$\begin{array}{c} {\text{i}}_{\text{t}}=\;\sigma ({\text{W}}_{\text{i}}\;.\;\left[{\text{h}}_{\text{t}-\text{1}},\;{\text{ x}}_{\text{t}}\right]+\;{\text{b}}_{\text{i}} \end{array}$$ (6)

The third gate is the output gate that determines which part of the updated cell state contributes to the hidden ${\text{h}}_{\text{t}}$ for the current time step.

The output layer is a dense layer with three units and a softmax activation function, designed to classify the EEG signals into three categories. This ensures that the output represents a probability distribution over the three classes.


$$\begin{array}{c} {\text{P}}_{\text{k}}=\;\frac{{\text{e}}^{{\text{z}}_{\text{k}}\;}}{\sum_{\text{j}=\text{1}}^{\text{3}}{\text{e}}^{{\text{z}}_{\text{j}}\;}} \end{array}$$ (7)

The same LSTM configuration was used for both CNN-LSTM.

### 2.8. Optimization and regularization

To optimize the training process and mitigate the risk of overfitting, following techniques were employed:

**Dropout:** Dropout layers were introduced (30–40%) after the LSTM blocks to limit overfitting. This method temporarily disables a proportion of units during training, which prevents the network from depending on specific activations and helps it generalize better to new EEG data [19].

**Adam Optimizer:** Model training was performed using the Adam optimizer. Adam combines momentum with adaptive learning rate adjustment, which makes convergence faster and more stable than conventional stochastic gradient descent [20].

**EarlyStopping:** EarlyStopping monitored the validation loss during training. If no improvement was observed for 15 consecutive epochs, training was stopped [21].

**ModelCheckpoint:** ModelCheckpoint was employed to automatically save the best-performing model during training based on validation loss, ensuring that the optimal model was preserved [18].

**ReduceLROnPlateau**: ReduceLROnPlateau reduced the learning rate by a factor of 0.5 after three epochs with no improvement in validation loss. This allows the model to fine-tune effectively during the later stages of training [22].

These steps stabilized training, limited fluctuations in accuracy and loss, and supported better generalization to new EEG data. Model performance was further assessed using LOSO (Leave one subject out), where each subject was held out once for testing. This evaluation was done only to assess subject-level generalization and model robustness not for the feature extraction during training. No manual feature engineering was performed, as spatial features were automatically learned by the convolutional layers while temporal dependencies were captured by the LSTM layers. The model was trained with a batch size of 32, and the dataset was shuffled to improve generalization. Although training was allowed for up to 50 epochs, early stopping usually ended the process earlier to reduce the risk of overfitting. The EEG data was reshaped to match the required (time_steps, features) format for the LSTM model.

## 3. Results

**Fig 5:**
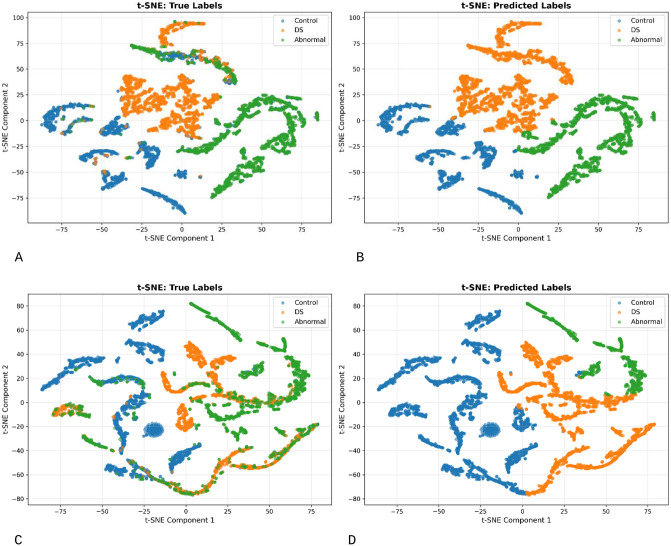
(A) t-SNE: Pre-trained (ShallowFBCSPNet) CNN-LSTM Model (B), (C) t-SNE: NON-Pretrained CNN-LSTM Model (D). t-SNE visualization of feature representations learned by pre-trained and non-pretrained CNN-LSTM models. (A) Two-dimensional t-SNE projection of feature embeddings extracted from the pre-trained (ShallowFBCSPNet-based) CNN-LSTM model, colored according to true class labels. (B) Corresponding t-SNE projection for the pre-trained model colored by predicted labels, showing close agreement with the true class structure. (C) t-SNE projection of feature embeddings from the non-pretrained CNN-LSTM model using true labels. (D) Predicted-label t-SNE projection for the non-pretrained model, illustrating reduced cluster compactness and increased overlap among classes. Overall, the pre-trained model exhibits more coherent and separable class representations compared with the non-pretrained counterpart.

**Fig 6:**
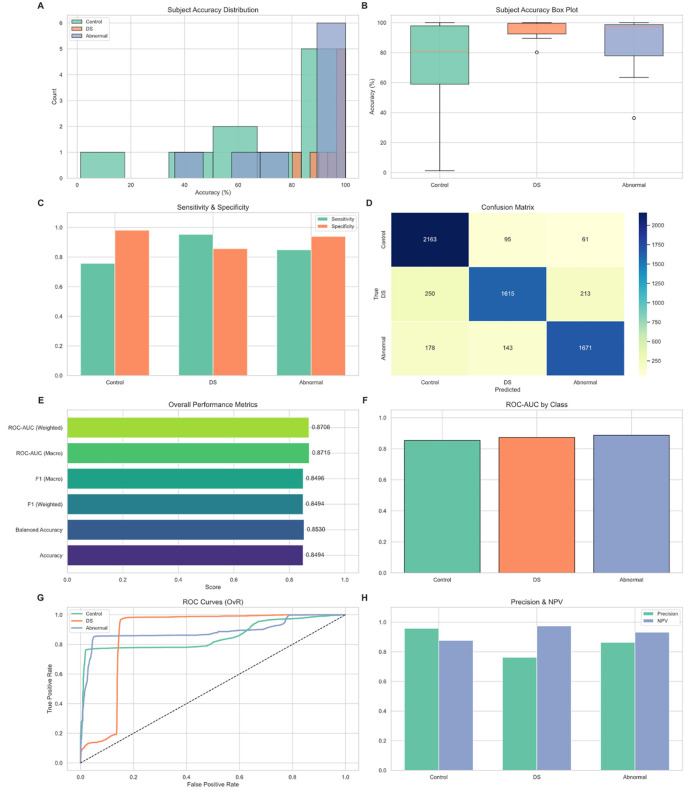
Summary of validation of the pre-trained CNN-LSTM model. This figure summarizes the performance of the pre-trained CNN-LSTM framework via pooled predictions of all subjects. (A) Subject-wise accuracy distributions of the Control, DS, and Abnormal groups, showing consistently high classification accuracy with limited variability across individual recordings. (B) Boxplots of subject accuracy for each class, giving a consolidated view of the central tendency and dispersion, highlighting high median accuracies and compact interquartile ranges. (C) Sensitivity and specificity values reported separately for each class, indicating strong and balanced detection capability across diagnostic groups. (D) Pooled confusion matrix shows distribution of correct and incorrect predictions across Control, DS, and Abnormal, showing a high proportion of correct classifications with relatively few misclassifications. (E) On a general scale of performance metrics such as overall accuracy, balanced accuracy, macro- and weighted F1-scores, and ROC–AUC, reflecting robust overall model performance. (F) Class-wise ROC–AUC scores indicating the high discriminative ability attained for each diagnostic group. (G) One-vs-rest ROC curves of all classes, indicating a favorable trade-off between true-positive and false-positive rates. (H) The precision and negative predictive value for each class state the high reliability of both positive and negative predictions made by the model.

**Fig 7:**
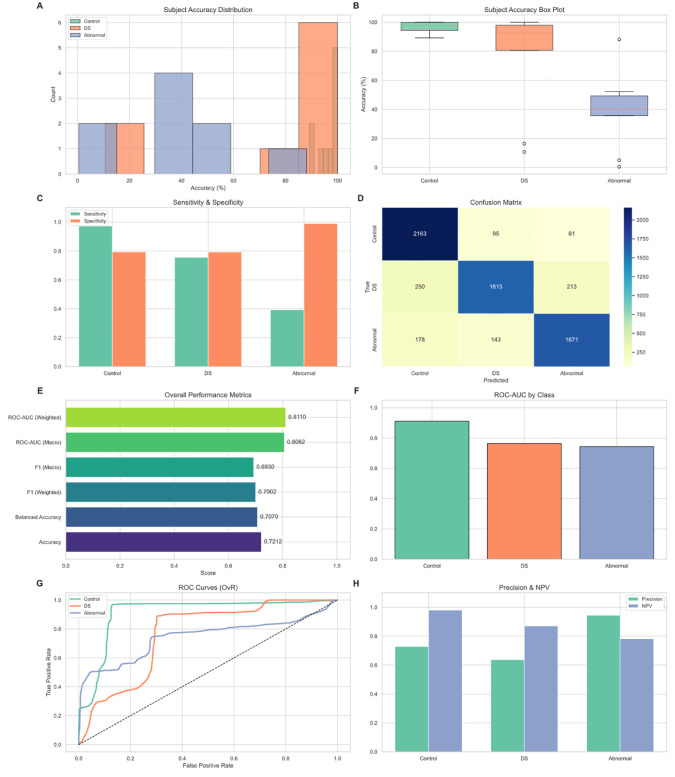
Performance of the non-pretrained CNN-LSTM model for multi-class EEG classification. (A) Subject-wise accuracy distribution for Control, DS, and Abnormal EEG classes, showing increased variability and lower overall accuracy compared with the pre-trained model. (B) Subject-level accuracy across classes shown in box plot, indicating reduced median accuracy, especially for Abnormal EEG. (C) Sensitivity and specificity across classes showing significant reductions in sensitivity for DS and Abnormal classes. (D) Confusion matrix of pooled LOSO predictions representing more interclass confusion between DS and Abnormal EEG. (E) Overall pooled performance metrics, namely accuracy, balanced accuracy, F1 scores, and ROC-AUC. (F) Class-wise ROC-AUC values. (G) One-vs-rest ROC curves demonstrating diminished separability relative to the pre-trained model. (H) Precision and NPV for each class. These results shows that training without pre-initialized EEG representations leads to less stable classification performance.

The performance of the proposed CNN-LSTM framework was evaluated using subject-level leave-one-subject-out (LOSO) cross-validation (Fig 5). For overall performance reporting, segment-level predictions from each test fold were aggregated (pooled) across subjects. A distinct performance difference was found between the pre-trained and non-pretrained CNN-LSTM models as shown in Table 3. The overall accuracy of the pre-trained model was 85%, with a balanced accuracy of 0.85, a weighted F1-score of 0.85, and a macro F1-score of 0.85 as shown in Fig 6. In contrast, the non-pretrained model resulted an accuracy of 0.72, balanced accuracy of 0.71, weighed F1-score of 0.70, and macro F1-score of 0.69, demonstrating reduced classification stability and generalization as shown in Fig 7. While the pretrained, model demonstrated high overall accuracy, subject-level performance showed notable variability, with a small subset of subjects exhibiting substantially reduced classification accuracy. These findings suggest that the pre-trained model achieved substantially higher discriminative capability (0.87 vs 0.81 macro AUC; 0.85 vs 0.72 accuracy), reflected by macro and weighted ROC-AUC values of 0.87, compared with 0.81 for the non-pretrained model as shown in Table 3.

**Table 3:** Performance comparison of the pretrained and non-pretrained CNN–LSTM models.

Metric	Pre-trained Model	Non-Pretrained Model
Overall Metrics		
Accuracy	0.85	0.72
Balanced Accuracy	0.85	0.71
F1-score (Weighted)	0.85	0.70
F1-score (Macro)	0.85	0.69
ROC–AUC (Macro)	0.87	0.81
ROC–AUC (Weighted)	0.87	0.81
Control Class		
Sensitivity	0.76	0.97
Specificity	0.98	0.79
Precision	0.96	0.73
NPV	0.88	0.98
ROC–AUC	0.85	0.91
Dravet Syndrome Class		
Sensitivity	0.95	0.76
Specificity	0.86	0.79
Precision	0.76	0.64
NPV	0.97	0.87
ROC–AUC	0.87	0.76
Abnormal Class		
Sensitivity	0.85	0.39
Specificity	0.94	0.99
Precision	0.86	0.94
NPV	0.93	0.78
ROC–AUC	0.89	0.74

Class-wise analysis (Tables 4 and 5) further highlighted the advantages of EEG-specific pretraining. For the Control, the pre-trained CNN-LSTM achieved 0.76 sensitivity and 0.98 specificity, with 0.96 precision, 0.88 negative predictive value, and 0.85 ROC-AUC as shown in Table 4. Although the non-pretrained model showed higher sensitivity for the Control class, this came at the cost of reduced specificity and poorer performance in DS and Abnormal classes as shown in Table 5. In the case of the DS, the pre-trained model demonstrated excellent detection ability, achieved a 0.95 sensitivity and 0.86 specificity with a precision of 0.76 and a negative predictive value of 0.97, and 0.87 ROC-AUC. For the abnormal class, pre-trained model results are similar robust values of 0.85 sensitivity, 0.94 specificity, 0.86 precision, 0.93 NPV, and 0.89 ROC-AUC. In comparison, the non-pretrained model exhibited pronounced reductions in sensitivity for both the DS (0.76) and Abnormal (0.39) classes, accompanied by increased inter-class confusion.

**Table 4:** Performance of the pre-trained CNN-LSTM model.

Subject	Class	Accuracy	Balanced Accuracy	F1 Weighted	F1 Macro	Segments
DS1	DS	95.47	0.95	0.98	0.33	318
DS2	DS	99.16	0.99	1.00	0.50	358
DS3	DS	100.00	1.00	1.00	1.00	434
DS4	DS	89.57	0.90	0.94	0.47	375
DS5	DS	99.15	0.99	1.00	0.33	415
DS6	DS	100.00	1.00	1.00	1.00	395
DS7	DS	92.45	0.92	0.96	0.48	328
DS8	DS	80.19	0.80	0.89	0.30	371
DS9	DS	99.58	1.00	1.00	0.50	246
A1a	Abnormal	100.00	1.00	1.00	1.00	259
A1b	Abnormal	97.41	0.97	0.99	0.33	434
A2a	Abnormal	98.71	0.99	0.99	0.33	184
A2b	Abnormal	63.48	0.63	0.78	0.26	367
A2c	Abnormal	99.17	0.99	1.00	0.50	310
A3a	Abnormal	90.85	0.91	0.95	0.48	322
A3b	Abnormal	36.36	0.36	0.53	0.18	264
A4	Abnormal	77.91	0.78	0.88	0.29	338
A5	Abnormal	97.96	0.98	0.99	0.49	330
CG1	Control	58.09	0.58	0.73	0.24	427
CG2	Control	100.00	1.00	1.00	1.00	319
CG3	Control	1.26	0.01	0.02	0.01	411
CG4	Control	96.61	0.97	0.98	0.33	468
CG5	Control	43.86	0.44	0.61	0.20	483
CG6	Control	100.00	1.00	1.00	1.00	415
CG7	Control	61.54	0.62	0.76	0.25	298
CG8	Control	88.46	0.88	0.94	0.31	336
CG9	Control	98.25	0.98	0.99	0.33	412
CG10	Control	73.42	0.73	0.85	0.28	280

**Table 5:** Subject-wise performance of the simple CNN-LSTM model.

Subject	Class	Accuracy	Balanced Accuracy	F1 Weighted	F1 Macro	Segments
DS1	DS	97.94	0.98	0.99	0.49	318
DS2	DS	99.58	1.00	1.00	0.50	358
DS3	DS	10.68	0.11	0.19	0.10	434
DS4	DS	92.61	0.93	0.96	0.32	375
DS5	DS	97.45	0.97	0.99	0.49	415
DS6	DS	100.00	1.00	1.00	1.00	395
DS7	DS	86.32	0.86	0.93	0.46	328
DS8	DS	80.66	0.81	0.89	0.30	371
DS9	DS	16.39	0.16	0.28	0.14	246
A1a	Abnormal	40.38	0.40	0.58	0.29	259
A1b	Abnormal	36.64	0.37	0.54	0.18	434
A2a	Abnormal	52.16	0.52	0.69	0.23	184
A2b	Abnormal	35.65	0.36	0.53	0.18	367
A2c	Abnormal	5.00	0.05	0.10	0.03	310
A3a	Abnormal	49.30	0.49	0.66	0.22	322
A3b	Abnormal	0.48	0.00	0.01	0.00	264
A4	Abnormal	43.78	0.44	0.61	0.30	338
A5	Abnormal	88.16	0.88	0.94	0.47	330
CG1	Control	94.19	0.94	0.97	0.49	427
CG2	Control	100.00	1.00	1.00	1.00	319
CG3	Control	98.74	0.99	0.99	0.50	411
CG4	Control	100.00	1.00	1.00	1.00	468
CG5	Control	96.49	0.96	0.98	0.49	483
CG6	Control	100.00	1.00	1.00	1.00	415
CG7	Control	89.23	0.89	0.94	0.31	298
CG8	Control	91.03	0.91	0.95	0.32	336
CG9	Control	99.75	1.00	1.00	0.50	412
CG10	Control	94.94	0.95	0.97	0.49	280

Evaluation on individual subjects showed that the pre-trained CNN-LSTM maintained largely high accuracy among most individual recordings. Several subjects under all three classes achieved near-perfect or perfect classification performance. However, notable exceptions included three Control subjects (CG1: 58%, CG3: 1.3%, CG5: 44%) and two Abnormal subjects (A2b: 63%, A3b: 36%) with substantially reduced accuracy. Additionally, one DS subject (DS8: 80%) showed relatively lower performance compared to other DS cases. These outliers likely reflect subject-specific signal characteristics or data quality variations., which likely indicates subject-specific variations in signals. Despite these outliers, the overall subject-level distribution demonstrated limited dispersion and stable central tendency, confirming the robustness of the pre-trained model across heterogeneous EEG recordings. The t-SNE visualizations shown in Fig 5 illustrate the feature distributions learned by the pre-trained and non-pretrained CNN-LSTM models for both true and predicted labels. While class overlap is observed in both cases, the figure provides a qualitative view that complements the quantitative performance results of using the CNN-LSTM model.

Overall, these results indicate that EEG signals from the DS class are more consistently distinguishable from Control and Abnormal EEG within the proposed framework. In addition, incorporating pre-trained EEG representations into the CNN-LSTM architecture leads to improved multi-class classification performance, greater subject-level consistency, and more clearly separable feature embeddings compared with training the same model from scratch.

Since performance metrics are computed using pooled segment-level predictions, subjects contributing a larger number of segments have a proportionally greater influence on overall metrics. The number of segments per subject shows moderate variation (mean ≈ 354; range: 184–483), with most subjects within ±23% of the mean, indicating no disproportionate influence from longer recordings. Additionally, the segment distribution across classes is reasonably balanced (DS: 32.7%, abnormal: 28.4%, control: 38.9%), supporting that the reported performance is not driven by subject- or class-level imbalance. Furthermore, the use of subject-independent LOSO evaluation ensures that all segments from a given subject are evaluated exclusively within the corresponding test fold, preventing data leakage and maintaining fair evaluation.

Performance comparison of the pre-trained and non-pretrained CNN-LSTM models for multi-class EEG classification using subject-level leave-one-subject-out (LOSO) validation. The pre-trained model achieved higher overall accuracy (0.85 vs. 0.72), balanced accuracy (0.85 vs. 0.71), and F1-scores (weighted: 0.85 vs. 0.70; macro: 0.85 vs. 0.69) compared with the non-pretrained model. Macro- and weighted ROC–AUC values were also higher for the pre-trained model (0.87) than for the non-pretrained model (0.81). Class-wise analysis shows that the pre-trained CNN-LSTM improved detection of Dravet Syndrome (sensitivity: 0.95 vs. 0.76; ROC–AUC: 0.87 vs. 0.76) and Abnormal (sensitivity: 0.85 vs. 0.39; ROC–AUC: 0.89 vs. 0.74), while having a high specificity across all the three classes. Although the non-pretrained model exhibited higher sensitivity for the Control class (0.97 vs. 0.76), this was Followed by reduced specificity (0.79 vs. 0.98). Overall, the results suggest that EEG-specific pretraining substantially improves subject-level generalization for this dataset and improves clinically relevant discrimination of pathological EEG patterns.

The table presents the classification performance across subjects as classified by the pre-trained CNN-LSTM model for the three categories Control, DS and Abnormal. The number of segments per subject varies due to differences in recording duration. However, LOSO evaluation ensures that all segments from each subject are evaluated independently. The measures include accuracy, balanced accuracy, weighted F1-score and consequently the macro F1-score, giving a comprehensive display of the differences among subjects. Generally, most subjects from all classes were able to achieve very high classification accuracy, many attaining almost perfect scores or indeed perfect scores. A very few subjects were particularly observed in the DS and Abnormal categories to be much lower than the rest, reflecting subject-specific variability most likely associated with signal heterogeneity or data quality disparity. Therefore, even with such outliers, the pre-trained model generally exhibits robust and consistent performance across nearly all subjects.

Table 5 indicates the performance across subjects of the simple CNN-LSTM model with no pretraining. While high accuracy appears in the control subjects, much variation across subjects in the DS or Abnormal categories is present such that many exhibits serious declines in both accuracy and F1 scores. The growing dispersion of results further strengthens the reduced robustness of the simple CNN-LSTM architecture compared to the pre-trained one about somewhat more challenging or heterogeneous subject recordings.

## 4. Discussion

DS is a severe infantile-onset epileptic and developmental encephalopathy that affects both genders, although the present cohort showed a higher proportion of female patients (58.3%). Seizures typically begin within the first 30 months of life, most commonly between 4 and 6 months, which aligns with the characteristic early onset of DS [2]. Previous studies have reported a median seizure onset age of approximately 5.7 months, ranging from 1.5 to 20.6 months [23]. This is consistent with our cohort findings, where the mean onset age was 7.04 months.

The hybrid CNN-LSTM framework showed generally consistent performance with some subject-specific variability in categorizing EEG recordings into Control, DS, and Abnormal groups under a subject-independent (LOSO) evaluation strategy. Using pre-trained architecture, the model attained an overall classification accuracy of approximately 85%, accompanied by closely aligned macro- and weighted-average precision and F1-scores, reflecting consistent class-wise performance across three categories. A multi-class AUC-ROC of around 0.87 further indicates that the framework can effectively distinguish between EEG patterns. Confusion matrix analysis showed that most of the recordings were correctly classified, with fewer misclassifications observed for the pre-trained model compared with the non-pretrained counterpart. The comparatively lower performance of the non-pretrained model is likely attributable to the limited number of subjects available for training, which constrains generalization under a leave-one-subject-out evaluation scheme. With an increase in subject diversity, improved performance from non-pretrained models may be expected, however, under the current dataset size and validation setting, EEG-specific pretraining appears to be a more effective strategy [24,25].

Compared with traditional machine learning approaches such as Support Vector Machines (SVM) and Random Forest, the hybrid CNN-LSTM framework is well suited to capturing both spatial and temporal dependencies in EEG signals. Random Forest-based methods have reported classification accuracies of approximately 88%, demonstrating reasonable performance when carefully engineered features are used [13], such approaches rely heavily on manual feature extraction and preprocessing choices. In contrast, CNN-LSTM architecture learns hierarchical representations directly from raw electroencephalogram data. This reduces the dependence on manual design of feature extraction, aiding in its effectiveness toward building a more general model for complex temporal EEG patterns.

From a translational perspective, it will be important to assess computational efficiency and resource requirements when deploying CNN-LSTM models in hospital environments with limited computational infrastructure [26]. Moreover, the clinical application will depend on its regulatory compliance with medical device requirements and data protection standards which are essential for safe and ethical healthcare implementation [27].

Additionally, the EEG data required preprocessing with Cz-referenced electrode configuration because it reduced muscle artifacts, which resulted in better signal quality than the standard monopolar configurations used in previous research studies [23]. Whereas earlier studies primarily relied on handcrafted EEG features, such as coherence and spectral power, this research employs an end-to-end deep learning model that automatically extracts relevant features, minimizing data loss. The pre-trained CNN-LSTM model achieved an AUC-ROC of approximately 0.87, demonstrates comparable discriminatory power compared to previous seizure classification models [13].

The model’s consistent classification performance, along with the distinct and compact clusters in the t-SNE visualization, suggests that DS is associated with distinct spatial and temporal EEG characteristics. These findings support but do not conclusively establish the presence of DS-associated EEG signatures, consistent with the disorder’s genetic and neurodevelopmental underpinnings [28]. A comparative analysis with prior studies indicates that pretrained CNN-LSTM model offers a more comprehensive approach, effectively integrating spatial and temporal feature extraction. For instance, some research [9] also reported a CNN-based deep learning model achieving high performance for EEG-based classification using subject-independent evaluation of subjects. Similarly, the proposed CNN-LSTM model demonstrates strong discriminative capability across three classes under a subject-independent setting, supporting its applicability for DS-focused EEG analysis.

Although there are still some misclassifications with subject-independent evaluation, further refinement of the model could enhance its diagnostic precision. The classification results will improve when additional genetic and neuroimaging data becomes available, delivering better insights into DS-related biomarkers. Future studies incorporating genetic and neuroimaging data alongside EEG may provide complementary information and support more comprehensive syndrome-level characterization [29]. Future work may investigate real-time deployment of the proposed framework in clinical settings, subject to further validation and optimization [9].

This study shows the significant role of deep learning in EEG analysis specifically for DS diagnosis, supporting improved diagnostic decision-making [30,31]. The approach may inform future systems aimed at continuous seizure monitoring in clinical and home environments [9]. Although this study does not directly address the treatment selection, the proposed framework may support future personalized epilepsy management by capturing patient-specific EEG patterns related to DS. With additional validation and the inclusion of treatment-response data, such EEG representations could help explore how brain activity patterns relate to the effectiveness of different antiepileptic drugs. The clinical EEG systems would benefit from this method because it decreases misdiagnosis rates while delivering better results for patients. By expanding on previous research and integrating new methodologies, this work underscores the transformative potential of AI in epilepsy diagnostics and treatment strategies [32].

The findings of this study should be interpreted as a proof-of-concept methodological investigation. The proposed framework was evaluated on a relatively small dataset without external validation or comparison to expert clinical interpretation. In addition, the abnormal EEG group is heterogeneous, which may introduce variability in classification performance. Therefore, while the results demonstrate the feasibility of automated EEG-based classification of DS-related patterns, they do not establish clinical diagnostic utility. Further studies using larger, multi-center datasets and prospective validation are required to assess generalizability and potential clinical applicability.

## 5. Conclusion

This study demonstrates that a hybrid CNN-LSTM model is effective for automatic classification of DS from EEG under a subject-independent evaluation. By jointly modeling the spatial and temporal EEG dependencies, the proposed method offers a strong alternative to previous conventional approaches that heavily rely on hand-crafted features in combination with classical machine learning classifiers. The utilization of pre-trained EEG representations additionally enhances classification stability and generalizability in the context with limited subjects, which emphasizes the advantage of representation learning for rare epilepsy syndromes such as DS.

Beyond diagnostic classification, this work explores EEG patterns associated with DS by identifying characteristic electroencephalography patterns observed in the dataset. Although in this work we focus on offline analysis, the results suggest that these models may be further investigated in future studies exploring their potential applicability in clinical decision-support settings with appropriate validation. Future research should aim to include larger and more heterogeneous multi-center cohorts to enhance generalizability, along with the incorporation of genetic or neuroimaging data to improve syndrome-level discrimination. The findings should be interpreted within the context of a relatively small dataset and the absence of external validation or comparison with expert clinical interpretation. After further refinement and clinical validation, the proposed deep learning-based EEG analysis supports the feasibility of automated EEG-based classification as a proof-of-concept methodological approach. Further validation is required before any clinical application.

## 6. Future directions

To further strengthen the methodological robustness and generalizability of the proposed framework, future work should involve larger and more diverse datasets collected across multiple clinical centers. Expanding the cohort would allow evaluation of model stability across heterogeneous patient populations, recording protocols, and EEG acquisition systems, which is essential for assessing reproducibility beyond a single-center setting. Subject-independent validation strategies should be emphasized to better account for inter-patient variability in EEG patterns and to reduce the influence of subject-specific features [33].

Another important direction is the prospective evaluation of the model under controlled clinical conditions. While the present study focuses on retrospective EEG analysis, future investigations could examine the feasibility of applying similar architectures to continuous or near–real-time EEG streams to assess temporal robustness and computational efficiency. Such studies would primarily serve as methodological validation rather than clinical deployment and would help clarify the practical constraints associated with real-world EEG data [34,35].

The research shows potential for growth through its integration of EEG data with other research techniques. The combination of electrophysiological signals with both genetic information and neuroimaging data such as fMRI creates a comprehensive method to identify disease-related abnormalities through neurophysiological and genetic and structural measurements. The multimodal approach enables researchers to create better feature representations, which will help upcoming studies at examine differences between DS and other epilepsy syndromes [36,37].

Finally, improving model interpretability remains a critical objective. Incorporating visualization techniques such as Grad-CAM or related attribution methods could help identify spatial and temporal EEG regions that can significantly contribute to classification decisions. Enhancing transparency at the electrode and time-window level would not only improve confidence in the learned representations but also provide neurophysiological meaningful insights that may guide future hypothesis-driven studies. Collectively, these directions aim to refine the framework as a reliable and interpretable deep learning methodology for EEG-based analysis rather than a standalone clinical diagnostic tool [38,39].

## 7. Challenges and limitations

The proposed CNN-LSTM framework shows promising results but there are several limitations which need to be acknowledged when evaluating the results. A primary challenge arises from the inherently limited availability of EEG data for DS, which constrained the number of subjects included in each diagnostic group. Although subject-independent validation was used to decrease subject-specific bias, the small cohort size limits statistical analysis power which affects performance estimate stability across new population samples [35,40]. Additionally, variation in the number of segments per subject may influence pooled metrics; however, subject-independent LOSO validation reduces potential bias from unequal segment contributions.

Another limitation relates to inter-subject and intra-subject variability in EEG recordings. Different factors such as seizure burden, developmental stage, medication status, and recording conditions leads to major differences in EEG patterns which particularly affects pediatric populations [41,42]. While the use of pre-trained EEG representations improved robustness under these conditions, residual variability remains difficult to fully capture with the available data and may contribute to inconsistent performance for certain individuals [43].

The study uses retrospective EEG recordings which were obtained through clinical protocols. As a result, the model’s behavior under continuous or real-world EEG monitoring scenarios, where noise levels, electrode displacement, and recording interruptions are more prevalent, was not assessed. This limits its conclusions in computational efficiency and temporal stability in operational environments [44].

Furthermore, although the pre-trained CNN-LSTM architecture demonstrated improved generalization compared with training from scratch, the pre-trained representations were derived from datasets with different clinical characteristics [45]. This domain mismatch may influence feature transferability and highlights the need for epilepsy-specific large-scale EEG pretraining resources [46].

Finally, the proposed framework focuses on classification performance without explicitly addressing interpretability at the neurophysiological level. While the model learns discriminative representations, the absence of systematic attribution analysis limits direct clinical insight into the specific EEG features driving classification decisions [47].

Accordingly, the present findings should be viewed as methodological validation rather than definitive clinical evidence. Future multi-center, prospective studies with larger datasets and enhanced interpretability are required to advance EEG-based deep learning methods for rare epilepsy syndromes such as DS [48,49].

## Abbreviation:

DS: Dravet Syndrome
SCN1A: Sodium Voltage-Gated Channel Alpha Subunit 1
Nav1.1: Voltage-Gated Sodium Channel Alpha Subunit Nav1.1
OMIM: Online Mendelian Inheritance in Man
EEG: Electroencephalogram
ADHD: Attention Deficit Hyperactivity Disorder
CNV: Copy Number Variant
WES: Whole Exome Sequencing
NGS: Next-Generation Sequencing
ACMG: American College of Medical Genetics and Genomics.
